# The effect of metal-containing nanoparticles on the health, performance and production of livestock animals and poultry

**DOI:** 10.1080/01652176.2022.2073399

**Published:** 2022-05-14

**Authors:** Izabela Michalak, Katarzyna Dziergowska, Mahmoud Alagawany, Mayada R. Farag, Nahed A. El-Shall, Hardeep Singh Tuli, Talha Bin Emran, Kuldeep Dhama

**Affiliations:** a Faculty of Chemistry, Department of Advanced Material Technologies, Wroclaw University of Science and Technology, Wroclaw, Poland; b Poultry Department, Faculty of Agriculture, Zagazig University, Zagazig, Egypt; c Forensic Medicine and Toxicology Department, Veterinary Medicine Faculty, Zagazig University, Zagazig, Egypt; d Department of Poultry and Fish Diseases, Faculty of Veterinary Medicine, Alexandria University, Edfina, El-Beheira, Egypt; e Department of Biotechnology, Maharishi Markandeshwar (Deemed to be University), Mullana, Ambala, Haryana, India; f Department of Pharmacy, BGC Trust University Bangladesh, Chittagong, Bangladesh; g Division of Pathology, ICAR-Indian Veterinary Research Institute, Izatnagar, Bareilly, Uttar Pradesh, India

**Keywords:** Nanoparticles, nanotechnology, livestock, poultry, health, performance

## Abstract

The application of high doses of mineral feed additives in the form of inorganic salts increases the growth performance of animals, but at the same, due to their low bioavailability, can contaminate the environment. Therefore, there is a need to find a replacement of administering high doses of minerals with an equally effective alternative. The application of lower doses of metal-containing nanoparticles with the same effect on animal production could be a potential solution. In the present review, zinc, silver, copper, gold, selenium, and calcium nanoparticles are discussed as potential feed additives for animals. Production of nanoparticles under laboratory conditions using traditional chemical and physical methods as well as green and sustainable methods – biosynthesis has been described. Special attention has been paid to the biological properties of nanoparticles, as well as their effect on animal health and performance. Nano-minerals supplemented to animal feed (poultry, pigs, ruminants, rabbits) acting as growth-promoting, immune-stimulating and antimicrobial agents have been highlighted. Metal nanoparticles are known to exert a positive effect on animal performance, productivity, carcass traits through blood homeostasis maintenance, intestinal microflora, oxidative damage prevention, enhancement of immune responses, etc. Metal-containing nanoparticles can also be a solution for nutrient deficiencies in animals (higher bioavailability and absorption) and can enrich animal products with microelements like meat, milk, or eggs. Metal-containing nanoparticles are proposed to partially replace inorganic salts as feed additives. However, issues related to their potential toxicity and safety to livestock animals, poultry, humans, and the environment should be carefully investigated.

## 1. Introduction

The inorganic salts, commonly used as feed additives exhibit poor bioavailability to animals, which is caused by ingredients that can inhibit absorption of micro- and macroelements (Scott et al. 2018; Matuszewski et al. 2020a; Alagawany and Abd El-Hack 2021; Górniak et al. 2022). Therefore, they may pose a threat to the environment through excretion of high mineral levels that can contaminate soil and aquatic environment (Mroczek-Sosnowska et al. 2015a, 2015b; Scott et al. 2018; Kociova et al. 2020; Matuszewski et al. 2020a; Cui et al. 2021; Szuba-Trznadel et al. 2021). It may be beneficial to apply solutions proposed by nanotechnology to minimize this negative effect on the environment, as well as to enhance health, performance and production of livestock animals and poultry.

Nowadays, nanobiotechnology is an emerging field in animal and veterinary sciences for a variety of practical applications such as therapeutic, diagnostic, and nutritional applications (Abd El-Hack et al. 2017; Prasad et al. 2018; Amlan and Lalhriatpuii 2020; El-Maddawy et al. 2022). Nanoparticles (NPs) of essential minerals, which size from 1 to 100 nm, could be used as an alternative to conventional forms of elements in animal diet (Mohamed et al. 2016; Swain et al. 2016; Scott et al. 2018; Abdollahi et al. 2020; Kociova et al. 2020; Szuba-Trznadel et al. 2021). It is assumed that much smaller doses of nanoparticles will be required to cover animal requirements for elements than bulk minerals (Vijayakumar and Balakrishnan 2014; Refaie et al. 2015; El Basuini et al. 2017; Scott et al. 2018; Youssef et al. 2019; Abdollahi et al. 2020; Szuba-Trznadel et al. 2021; Ouyang et al. 2021) and thus the environmental impact caused by the high concentration of inorganic salts will be alleviated (Vijayakumar and Balakrishnan 2014; Ouyang et al. 2021). Reduction in the quantity of minerals supplemented to animal diet could reduce the feed cost as well (Vijayakumar and Balakrishnan 2014). Additionally, nano-forms of elements can increase bioavailability to animals (Vijayakumar and Balakrishnan 2014; Hill and Li 2017; Youssef et al. 2019; Hidayat et al. 2021), due to their properties such as small size, good homogeneity, high surface area and physical reactivity (Grodzik and Sawosz 2006; Scott et al. 2018; Abdollahi et al. 2020; Amlan and Lalhriatpuii 2020; Ouyang et al. 2021). The biological properties of nanoparticles such as lesser dose, lower antagonism, greater absorption rate and better tissue distribution can also be beneficial to animals (Amlan and Lalhriatpuii 2020). It is well-known that nanoparticles have a great potential even at very low doses (Matuszewski et al. 2020a).

Nanoparticles in animal nutrition are explored for growth performance, feed utilization and health status (Abd El-Hack et al. 2017; Amlan and Lalhriatpuii 2020; Dawood et al. 2021). Nano-forms of micro- and macroelements most frequently increase the body weight, average daily gain, and improve the feed conversion ratio (FCR) (Bąkowski et al. 2018; Yusof et al. 2019). NPs are used to cover the animal's demand for elements, improve their productivity, ameliorate microbial profile and immune status, as well as reduce the risk of diseases. Nanoparticles are known for their antibacterial, antifungal, antiviral, antiprotozoal, antioxidative properties, etc. Silver, copper, selenium, and zinc nanoparticles can constitute alternative health and growth promoting additives to antibiotics (Sawosz et al. 2007; Pineda et al. 2012; Bąkowski et al. 2018; Kumar and Bhattacharya 2019; Yusof et al. 2019; Nabi et al. 2020; Sheiha et al. 2020; Hidayat et al. 2021; Morsy et al. 2021; Ouyang et al. 2021; El-Maddawy et al. 2022). Nano-copper and nano-zinc can also increase the activity of the superoxide dismutase enzyme, which constitutes a very important antioxidant defense against oxidative stress (Gonzales-Eguia et al. 2009; Refaie et al. 2015; Hidayat et al. 2021). Nano-selenium is also known to increase the activity of this enzyme (Kojouri et al. 2020), and improve the efficiency of the antioxidant system and prevent oxidative stress (Shi et al. 2011a; Sadeghian et al. 2012; El-Deep et al. 2016; Kojouri et al. 2020; Han et al. 2021).

However, it should be remembered that small size of nanoparticles might affect their toxicity by increasing the cellular uptake and translocation in the animal’s body (Gatoo et al. 2014; Scott et al. 2016). At the cellular level, nanoparticles can cause inflammation or even cell death (Bąkowski et al. 2018). Supplementation of nanoparticles to the animal diet can induce pathological changes in animal tissues, like liver, pancreas, kidney, small intestine, adrenal glands and brain (Bąkowski et al. 2018). Therefore, detailed research is still required to confirm the safety of application of metal-containing nanoparticles in animal nutrition, avoiding any harm to livestock, the environment, and human beings (Scott et al. 2018).

This comprehensive review aims to provide an up-to-date knowledge about the production of metal/metal oxides nanoparticles (zinc (Zn), silver (Ag), copper (Cu), gold (Au), selenium (Se), and calcium (Ca)) serving as feed additives and their application in the nutrition of various animal species (e.g., poultry, pigs, ruminants, and rabbits). Based on the literature mainly from the last decade, the effect of nano-minerals on livestock and poultry health, performance and production will be discussed. The potential harmful effect of metal/metal oxide nanoparticles on animals will also be highlighted.

## 2. Production of nanoparticles for animal research

Many minerals are used as nanoparticles in animal research. These include aluminium (Li et al. 2011), calcium (Matuszewski et al. 2020a; Abdelnour et al. 2021; Abo El-Maaty et al. 2021), copper (Gonzales-Eguia et al. 2009; Miroshnikov et al. 2015; Mroczek-Sosnowska et al. 2014, 2015a, 2015b, 2017; Refaie et al. 2015; Joshua et al. 2016; Ognik et al. 2016b; Scott et al. 2016; El Basuini et al. 2017; Tomaszewska et al. 2017; Scott et al. 2018; Aminullah et al. 2021; Morsy et al. 2021; Naz et al. 2021), gold (Sembratowicz et al. 2016; Sanati et al. 2019; Hassanen et al. 2020), iron (Pilaquinga et al. 2021), magnesium (Kesmati et al. 2016; Mazaheri et al. 2019; Abdelnour et al. 2021), nickel (Gong et al. 2016), selenium (Zhang et al. 2001, 2008; Shi et al. 2011a; Wu et al. 2011; Sadeghian et al. 2012; Xun et al. 2012; El-Deep et al. 2016; Joshua et al. 2016; Muralisankar et al. 2016; Yaghmaie et al. 2017; Gangadoo et al. 2018, 2020; Hassan et al. 2020; Kojouri et al. 2020; Nabi et al. 2020; Sheiha et al. 2020; Han et al. 2021; Rana, 2021), silver (Grodzik and Sawosz 2006; Sawosz et al. 2007; Fondevila et al. 2009; Fondevila, 2010; Ahmadi and Rahimi 2011; Pineda et al. 2012; Kout-Elkloub et al. 2015; Ognik et al. 2016a; Conine and Frost 2017; Bąkowski et al. 2018; Abdelsalam et al. 2019; Kumar and Bhattacharya 2019; Dung et al. 2020; Abdelnour et al. 2021; Awaad et al. 2021; Bidian et al. 2021; Niemiec et al. 2021), titanium (Li et al. 2011) and zinc (Joshua et al. 2016; Swain et al. 2016; Hassan et al. 2017; Olgun and Yildiz 2017; Bąkowski et al. 2018; Othman et al. 2018; Bakhshizadeh et al. 2019; Yusof et al. 2019; Abdollahi et al. 2020; Kociova et al. 2020; Cui et al. 2021; Eskandani et al. 2021; Hidayat et al. 2021; Mahmoud et al. 2021; Ouyang et al. 2021; Szuba-Trznadel et al. 2021; El-Maddawy et al. 2022) nanoparticles.

**Figure 1.**
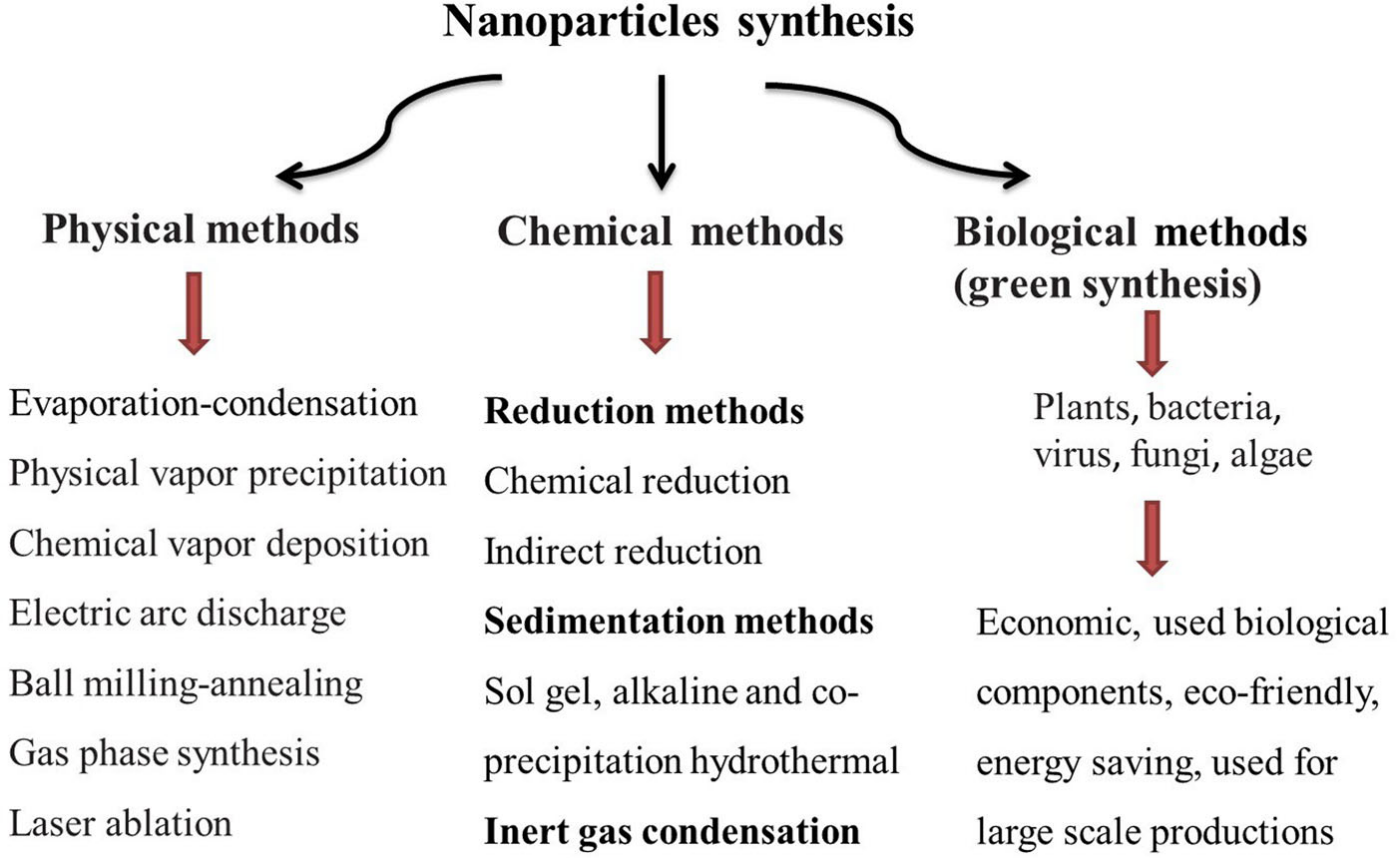
Manufacturing methods for nanoparticles synthesis.

These nanoparticles can be synthesized in the laboratory or are commercially available. Nanoparticles can be produced using traditional chemical and physical methods, as well as green and sustainable method – biosynthesis with plant extracts or microorganisms (Gopi et al. 2017; Yusof et al. 2019; Abdelnour et al. 2021). The general scheme showing available NPs production methods is presented in Figure 1.

There are several examples of laboratory-made mineral nanoparticles, which were tested as feed additives. They include the following types Ag (Ognik et al. 2016a; Abdelsalam et al. 2019; Dung et al. 2020; Kumar and Bhattacharya 2019; Awaad et al. 2021), Au (Sembratowicz et al. 2016; Hassanen et al. 2020), Ca (Abo El-Maaty et al. 2021), Cu (Gonzales-Eguia et al. 2009; Miroshnikov et al. 2015; Refaie et al. 2015; Joshua et al. 2016; Ognik et al. 2016b; Morsy et al. 2021), Se (Sadeghian et al. 2012; Joshua et al. 2016; Gangadoo et al. 2018; Gangadoo et al. 2020; Sheiha et al. 2020), and Zn (Joshua et al. 2016; Bakhshizadeh et al. 2019; Kociova et al. 2020; Hidayat et al. 2021; Mahmoud et al. 2021; Ouyang et al. 2021).

### 2.1. Physical methods

Physical methods include evaporation–condensation, chemical vapor deposition, laser ablation, electric arc discharge, ball milling–annealing, gas phase synthesis and physical vapor precipitation. The biggest advantages of these methods are the absence of solvent contamination and the maximal recovery of nanoparticles (Patra and Lalhriatpuii 2020; Abdelnour et al. 2021).

Gonzales-Eguia et al. (2009) prepared copper nanoparticles using the wet polish method with a ball grinding machine. These nanoparticles were applied as a supplementation for weanling pigs. Miroshnikov et al. (2015) produced copper nanoparticles for chicken by high temperature condensation. Furthermore, copper nanoparticles injected to chicken eggs were manufactured by a non-explosive high voltage method (Mroczek-Sosnowska et al. 2015a, 2015b, 2017; Scott et al. 2016). Joshua et al. (2016) prepared copper nanoparticles using electrochemical method with copper rods as anode and cathode. Ognik et al. (2016b) used copper nanoparticles prepared in a physical process – a non-explosive, high-current method for degradation of metals – as additives to water of chickens. Most recently, Aminullah et al. (2021) used induction coupled plasma method to prepare copper nanoparticles that were used as a supplement for Swarnadhara breeder hens. Sembratowicz et al. (2016) used in chicken feeding gold nanoparticles also produced by a non-explosive, high-current method for degradation of metals. Sawosz et al. (2007) applied, in their research on quail, silver nanoparticles (added to the drinking water) produced by the solid–liquid phase discharge method. Pineda et al. (2012) used in broiler chickens feed silver nanoparticles also produced by a non-explosive high voltage method (Polish Patent 3883399) from high purity metals and demineralized water. Mahmoud et al. (2021) checked if zinc oxide (ZnO) nanoparticles, prepared by mechanical milling of a commercial ZnO with a planetary ball mill (Othman et al. 2018), can prevent multi-drug resistant *Staphylococcus*-induced footpad dermatitis in broilers. In summary, physical methods are most often used to produce copper nanoparticles that were mainly applied as feed additives for poultry.

### 2.2. Chemical methods

Chemical methods include chemical reduction, sol gel method and inert gas condensation. They allow achieving more effective and controlled bulk production of nanoparticles as compared to physical methods. The disadvantage is possible toxicity due to the use of hazardous chemicals during the synthesis (Patra and Lalhriatpuii 2020; Abdelnour et al. 2021).

Joshua et al. (2016) used chemical synthesis with starch as a stabilizing agent to produce ZnO nanoparticles, the effect of which on post-hatch performance of broiler chickens was investigated. Most recently, zinc nanoparticles, used as a supplement to broiler chicken's diet, were synthesized using nano-chelating technology (Eskandani et al. 2021). Bakhshizadeh et al. (2019) prepared zinc nanoparticles for dietary cows using the chemical co-precipitation method with zinc nitrate and sodium hydroxide. Kociova et al. (2020) synthesized zinc phosphate-based nanoparticles for feeding of weaned piglets in two ways – the first one was from zinc nitrate and diammonium phosphate and the second one from zinc nitrate and tetrasodium pyrophosphate. Li et al. (2021) used the sol–gel process to create ZnO quantum dots (ZnO QDs) with a size range of 3–6 nm and studied their antibacterial properties *in vitro* and *in vivo* (chickens).

Ognik et al. (2016a) synthesized silver nanoparticles for chicken feeding by reducing silver ions with trisodium citrate at 100 °C. Abdelsalam et al. (2019) prepared silver nanoparticles from sodium tricitrate aqueous solution and silver nitrate and the colloid form of nanoparticles was injected to rabbits. For the synthesis of silver nanoparticles for poultry, Kumar and Bhattacharya (2019) used hydrothermal method with an autoclave (to the mixture of polyvinylpyrrolidone and sodium borohydride, aqueous solution of silver nitrate was added). Silver nanoparticles synthesized via chemical reduction method, using silver nitrate and sodium borohydride as a reducing agent, as well as chitosan solution as a stabilizer, were applied as antiviral agents against African swine fever virus (Dung et al. 2020). Awaad et al. (2021) also used chemical reduction method, in which silver nitrate and aqueous solutions containing acetyl trimethyl ammonium bromide and hydrazine hydrate were applied to receive silver nanoparticles, supplemented to broiler chickens experimentally infected with *Escherichia coli*.

Refaie et al. (2015) prepared copper nanoparticles from copper nitrate trihydrate and ethylene glycol and used them as a supplement for rabbits. Morsy et al. (2021) synthesized copper oxide nanoparticles as chicken's feed additives via chemical precipitation method, using copper chloride dihydrate and sodium hydroxide solutions in ethanol. Chemical synthesis of copper oxide nanoparticles in the research of Naz et al. (2021) was achieved by the reaction of copper acetate monohydrate with sodium hydroxide upon continuous stirring and heating. The aim of this work was to examine the copper NPs toxicity on rats and offspring. Muralisankar et al. (2016) used chemical synthesis with copper chloride, sodium dodecyl benzenesulfonate and hydrazine hydrates to prepare copper nanoparticles, which were used as supplementation on freshwater prawn *Macrobrachium rosenbergii* post larvae.

Selenium nanoparticles were chemically produced from sodium selenite and glutathione by Zhang et al. (2001) and later used as a chemo-preventive agent for mice (Zhang et al. 2008). Later, Sadeghian et al. (2012) produced selenium nanoparticles from selenium oxide and ascorbic acid and used them as a feed additive for sheep. Joshua et al. (2016) used chemical synthesis with selenium powder and sodium hydroxide to prepare nanoparticles for broiler chickens’ supplementation. Gangadoo et al. (2018, 2020) synthesized selenium nanoparticles for poultry using chemical reduction with selenium tetrachloride, ascorbic acid as a reducing agent and polystyrene-4-sulfonate as a protecting agent. Recently, Hassan et al. (2020) used sodium sulphate and selenium powder to produce selenium nanoparticles for broiler chickens exposed to heat stress. Selenium nanoparticles were also chemically synthesized by Kojouri et al. (2020), who used ascorbic acid solution and aqueous solution of selenium oxide and checked their impact on neonatal lamb weight gain pattern. Sheiha et al. (2020) used the selenium nanoparticles prepared by wet chemical approach using sodium selenite and l-cysteine as reagents in the feeding of growing rabbits reared under thermal stress.

Hassanen et al. (2020) synthesized gold nanoparticles for broiler chickens by chemical reduction of gold chloride hydrate with tri-sodium citrate dehydrate under boiling conditions. Iron (II and III) oxide nanoparticles were synthesized using a co-precipitation method and were used to check their effect on fertility and iron bioaccumulation in *Drosophila melanogaster* (Pilaquinga et al. 2021). Based on the presented examples, chemical methods are much more often used for the synthesis of a wide range of nanoparticles (with Zn, Ag, Cu, Se, Au, Fe) than physical methods and these NPs have potential applications as feed additives in the nutrition of many animal species, such as chickens, cows, pigs, piglets, rabbits, sheep, etc.

### 2.3. Biological methods

Biological methods are currently gaining popularity because they are eco-friendly, efficient, easy, and less toxic than traditional methods. In this method, plant extracts, algal extracts, or microorganisms (bacteria, viruses, and fungi) are used as simple substitutes for chemical and physical processes (Eszenyi et al. 2011; Patra and Lalhriatpuii 2020; Abdelnour et al. 2021). The compounds in these extracts such as polyphenols, terpenoids, sugars, proteins, etc. act as reducing agents maintaining the minerals in a reduced state during biosynthesis (Gopi et al. 2017). The limitations for biological methods are maintaining the culture media and the culture conditions, the difficulty in nanoparticle's recovery, and the duration in the creation of nanoparticles (Abdelnour et al. 2021). These methods of synthesis are currently not as popular as physical or chemical, but their popularity has grown in the last few years.

For the biological synthesis of calcium carbonate nanoparticles, serving as a supplementation for aged laying hens, Abo El-Maaty et al. (2021) used extract from brown macroalga *Sargassum latifolium*. Naz et al. (2021) prepared copper oxide nanoparticles from *Rhus punjabensis* leaf extract and compared their toxicity during pregnancy and lactation in rats and offspring with chemically produced nanoparticles. El-Deep et al. (2016) used biosynthesized selenium nanoparticles (with the use of bacteria strains like *Lactobacillus casei*, *Streptococcus thermophilus*, *Bifidobacterium* BB-12, *Lactobacillus acidophilus* (LA-5), and *Lactobacillus helveticus* (LH-B02)) in the feeding of broiler chickens. Sheiha et al. (2020) also used lactic acid bacteria isolated from human breast milk to biosynthesize selenium nanoparticles. Bidian et al. (2021) biosynthesized silver nanoparticles with the use of *Viburnum opulus* fruit extract to check their impact on the ultrastructure and cell death in the testis of offspring rats. Hidayat et al. (2021) used guava leaf extract (*Psidium guajava*) to biosynthesize zinc nanoparticles that were used as a supplementation of broiler chicken’s diet. Despite many advantages of nanoparticles biosynthesis, the variability of organisms that are used for their direct synthesis, as well as the production of natural extracts rich in bioactive compounds serving as reducing/stabilizing agents of nanoparticles should be considered. Additionally, unlike physical and chemical methods used for nanoparticles production, large-scale research is lacking in NPs biosynthesis.

### 2.4. Commercially available nanoparticles

A wide range of nanoparticles, which could be used in animal studies, is accessible on the market as shown in numerous publications, for example Ag (Grodzik and Sawosz 2006; Pineda et al. 2012), Au (Sembratowicz et al. 2016), Cu (Mroczek-Sosnowska et al. 2015b; Mroczek-Sosnowska et al. 2017; Aminullah et al. 2021), Se (Shi et al. 2011a; Xun et al. 2012; Yaghmaie et al. 2017; Han et al. 2021), and Zn (Olgun and Yildiz, 2017; Abdollahi et al. 2020; Cui et al. 2021; Eskandani et al. 2021; Szuba-Trznadel et al. 2021; El-Maddawy et al. 2022). In many experiments on animals and poultry, nanoparticles were purchased to check their impact on performance, productivity, health, and disease prevention and control. Han et al. (2021) used selenium nanoparticles as a supplementation for lactating dairy cows; Shi et al. (2011a) and Xun et al. (2012) used selenium nanoparticles to check their impact on feed digestibility, rumen fermentation and urinary purine derivatives in sheep; selenium nanoparticles were tested by Wu et al. (2011) on cashmere goats; Yaghmaie et al. (2017) used selenium nanoparticles in experiments on lambs; Grodzik and Sawosz (2006) used, in their research on chicken embryo development, silver nanoparticles; Szuba-Trznadel et al. (2021) checked the influence of zinc nanoparticles on growth performance and zinc status of weaned piglets; Abdollahi et al. (2020) used ZnO nanoparticles in feeding experiments on young Holstein calves to examine performance, rumen fermentation, mineral status, and antioxidant activity; Ouyang et al. (2021) used ZnO nanoparticles to evaluate their antimicrobial properties in weanling piglets and El-Maddawy et al. (2022) used zinc oxide nanoparticles to investigate their anticoccidial efficacy in broiler chickens infected with mixed *Eimeria* species in an experimental setting.

## 3. Applications of nanoparticles as feed additives on animal and poultry performance and health

**Figure 2.**
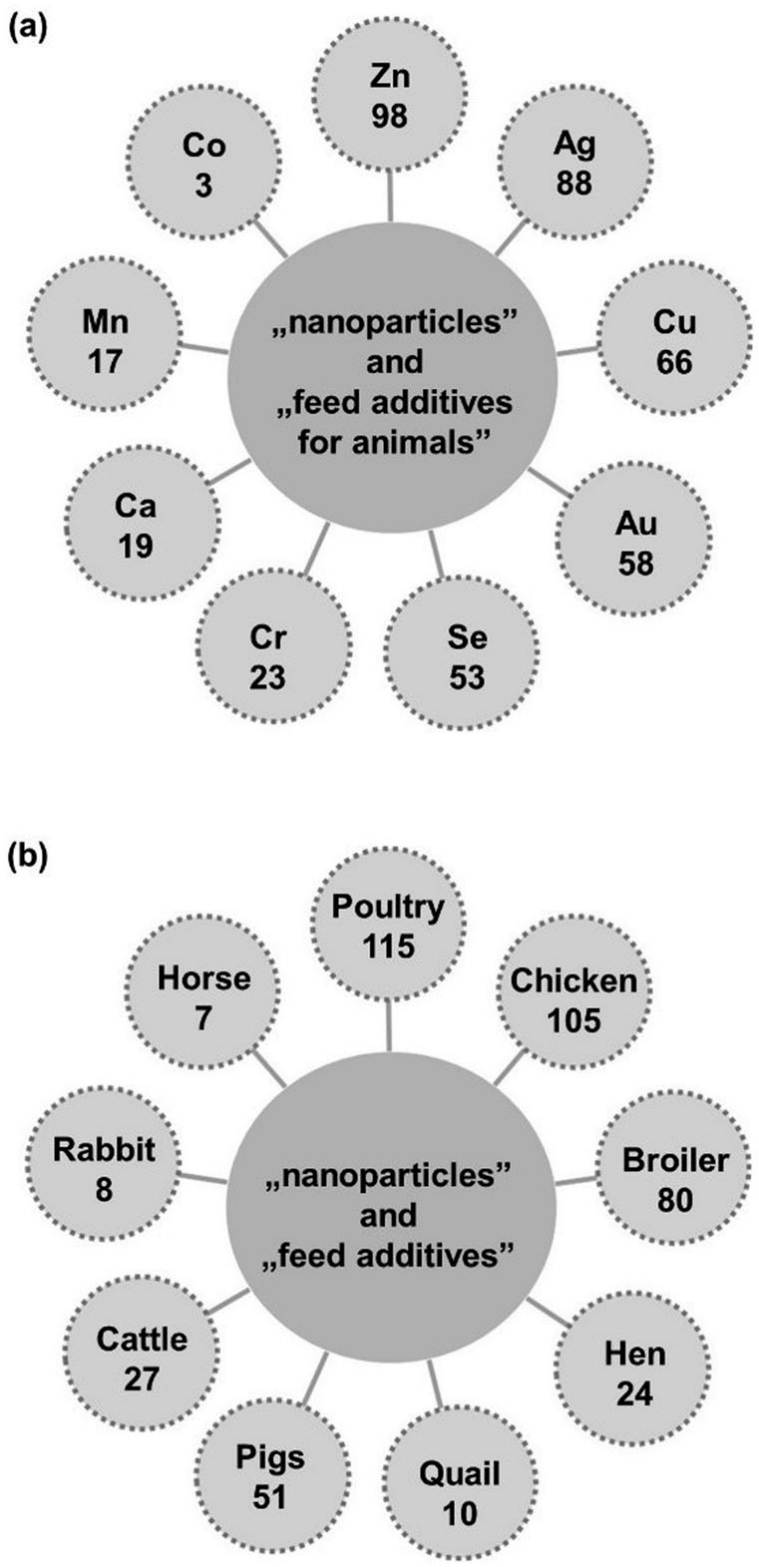
The number of papers on (a) type of nanoparticles used as feed additives for animals and (b) the species of animal supplemented with nanoparticles as feed additives (Web of Science, September 9, 2021).

Different types of metal/metal oxide nanoparticles have already been tested as potential feed additives. Figure 2 presents the number of papers, according to the Web of Science database, on (a) type of nanoparticles used as feed additives for animals and (b) the species of animal supplemented with nanoparticles as feed additives. The most often tested are zinc, silver, and copper nanoparticles and among animal species the application of nanoparticles in poultry diet dominates (Mohapatra et al. 2014a,b; Abd El-Hack et al. 2017).

In the first step of testing metal nanoparticles as potential animal feed additives, many researchers conduct preliminary nano-toxicological studies to determine the impact of examined nanoparticles on cell cultures (Leng et al. 2020) or animal models, for example mice (Zhang et al. 2008; Kesmati et al. 2016), rats (Tomaszewska et al. 2017; Mazaheri et al. 2019; Sanati et al. 2019; Kociova et al. 2019; Bidian et al. 2021; Naz et al. 2021), worms (Ayech et al. 2020), insects like *Drosophila melanogaster* (Pilaquinga et al. 2021) or in the case of aquatic organisms on water flea, like *Daphnia magna* (Gong et al. 2016; Conine and Frost 2017), *Ceriodaphnia dubia* (Li et al. 2011) or fish (Gutierrez et al. 2021). The findings obtained for these organisms may be extrapolated on other living beings (Pilaquinga et al. 2021).

**Figure 3.**
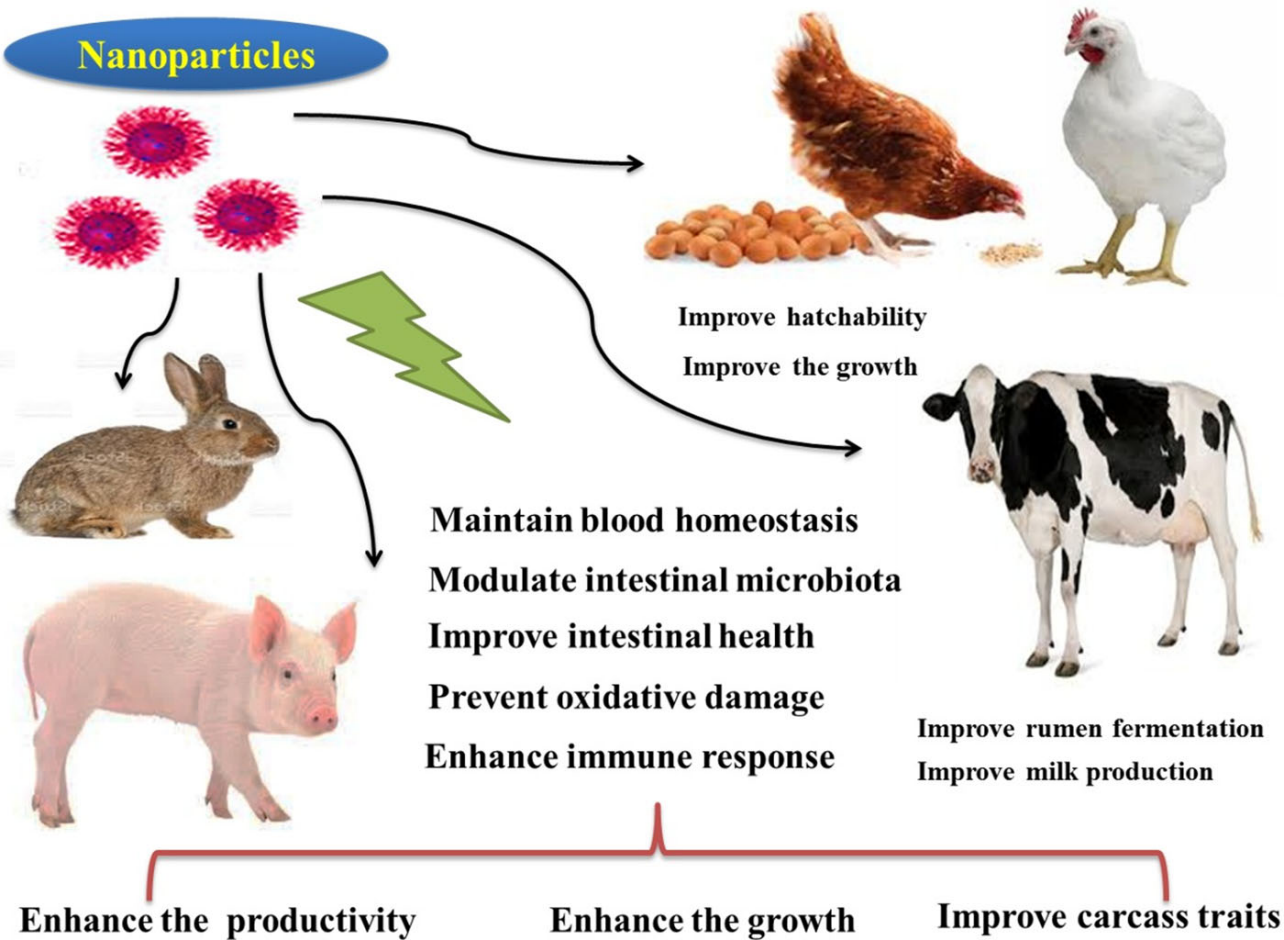
Beneficial effects of nanoparticles on animal health and performance.

In this review we focus on the impact of the metal-containing nanoparticles on poultry, pigs, ruminants, and rabbits. The beneficial effects of nano-minerals on animals are generally illustrated in Figure 3.

When using nanoparticles in animal nutrition, several aspects seem to be important. Metal/metal oxide nanoparticles used as feed additives can be supplemented directly to the feed or to the drinking water. In the case of poultry, *in ovo* administration of nanoparticles can be used as a method of nano-nutrition, supplying the embryos with an additional amount of nutrients (Pineda et al. 2012; Mroczek-Sosnowska et al. 2014, 2015b, 2017; Joshua et al. 2016; Saeed et al. 2019). It is hypothesized that *in ovo* supplementation of metal-containing nanoparticles allows for better utilization of microelements during embryo development than in the case of feed supplementation (Mroczek-Sosnowska et al. 2015b). Silver nanoparticles administered *in ovo* may also constitute potential antimicrobial growth-promoting supplement for poultry (Grodzik and Sawosz 2006; Pineda et al. 2012). Another method of administering metal-containing nanoparticles is parenteral injection – intramuscular (e.g., chicken – Miroshnikov et al. 2015) or subcutaneous (e.g., rabbits – Abdelsalam et al. 2019).

As seen in Table 1, in the feeding experiments, the effect of nanoparticles on animals is usually compared with organic or more frequently inorganic forms of elements (e.g., Gonzales-Eguia et al. 2009; Sadeghian et al. 2012; Xun et al. 2012; Mroczek-Sosnowska et al. 2015b; El-Deep et al. 2016; Olgun and Yildiz 2017; Yaghmaie et al. 2017; Gangadoo et al. 2018; Bakhshizadeh et al. 2019; Abdollahi et al. 2020; Aminullah et al. 2021; Eskandani et al. 2021; Hidayat et al. 2021; Ouyang et al. 2021; Szuba-Trznadel et al. 2021). For example, interesting results were presented by Sheiha et al. (2020) who compared the effect of two types of selenium-nanoparticles – biologically or chemically-synthesized on rabbits exposed to thermal stress. Better effects in terms of growth, carcass characteristics, oxidative and inflammatory parameters were observed for biosynthesized NPs.

**Table 1.** Effects of nano-minerals on growth performance and productivity of different animal species (available literature from the last 10 years).

Type of NPs	Dose and (size of NPs)	Animals and age	Effects and health benefits	References
ZnNPs	30, 50, 70 and 90 mg/kg (<50 nm)	Broiler chicken (1–42 days old)	Improvement of performance, carcass characteristics, humoral immunity, meat quality and Zn content in meat for 70 mg/kg	Eskandani et al. (2021)
ZnNPs	45, 90, 135 and 180 mg/kg	Broilers (1–42 days old)	Up to 90 mg ZnNPs/kg – a positive effect on performance, antioxidant activity	Hidayat et al. (2021)
ZnNPs	0.1, 0.2, 0.3 and 0.4 g/kg	Growing Japanese quails	Doses of 0.1–0.3 g/kg increased ALT, AST, LDH, SOD, GPX, MDA, IgG and IgM activities; a significant increase in growth for 0.2 g/kg of ZnNPs	Reda et al. (2021)
ZnONPs	60 and 30 mg/kg (30 nm)	Laying hens (55–56 weeks old)	Improved egg production, phagocytic activity and index, serum SOD and GSH-Px activities	El-Katcha et al. (2018)
ZnONPs	50, 75 and 100 mg/kg (35–45 nm)	Laying hens (42–54 weeks old)	Negative impact on eggshell thickness and bone mechanical properties	Olgun and Yildiz (2017)
ZnONPs	40 mg/kg (27 nm)	Broiler chickens (1–42 days old)	Improved the overall performance, Zn content in blood	Badawi et al. (2017)
Nano-Zn	20, 40, 60 and 80 µg/egg (< 100 nm)	Broiler chickens *in ovo* (18^th^ day of incubation)	No deleterious effect on the developing embryo and percentage of hatchability	Joshua et al. (2016)
ZnONPs	40 mg/kg (39.2–41.3 nm)	Broiler chickens (1–35 days old)	Improved performance (body weight gain, feed efficiency) and gut health (villus height and crypt depth)	Hafez et al. (2017)
ZnONPs	50 mg/kg (27 nm)	Broiler chickens (1–42 days old)	Increased SOD activity and decreased MDA; increased content of Fe and Cu in the hepatic tissue and content of Zn in the tibia; positive effect on mRNA expression of insulin like growth factor-1 and growth hormone genes	Ibrahim et al. (2017)
ZnONPs	40 and 80 mg/kg (19.3 nm)	Broiler chickens (1–35 days old)	Improved gut health by increasing villus height, and villus surface area of broiler small intestine	Ali et al. (2017)
Nano-Zn	15, 30 and 60 mg/kg	Giriraja chickens (1–56 days old)	Improved growth rate, increase in body weight, feed consumption ratio as compared to zinc sulfate for 60 mg/kg	Pathak et al. (2016)
Nano-Zn	40, 60 and 80 mg/kg	Broiler chickens (1–42 days old)	Improved performance – body weight gain, feed intake and feed conversion ratio and immune response under heat stress conditions especially for 80 mg/kg as compared to zinc sulfate	Sagar et al. (2018)
ZnONPs/egg	0.04 and 0.08 mg (< 100 nm)	Broiler chickens *in ovo* (18^th^ day of incubation)	No effect on performance parameters; no effect on immune response; reduction in percentage of hatchability	Jose et al. (2018)
ZnONPs	100 and 200 mg/kg	Broiler chickens (1–21 days old)	Increased carcasses yield; increased weight of lymphoid and digestive organs during the starter stage	Mohammadi et al. (2015)
Nano-Zn	0.3, 0.06 and 0.03 mg/kg	Broiler chickens (1–42 days old)	Improved health (immunity) status and increase in Zn concentration in tibia bone, liver, and blood serum for 0.06 mg/kg	Sahoo et al. (2014)
ZnONPs	30, 60, 90 and 120 mg/kg (40 nm)	Broiler chickens (1–21 days old)	Improved antioxidant parameters and serum enzymes activity during the starter period	Ahmadi et al. (2014)
ZnONPs	500, 1000, 2000 and 3000 mg/kg	Piglets	Anti-diarrhoea effect, improved growth when compared with ordinary-size ZnO (3000 mg/kg)	Ouyang et al. (2021)
ZnONPs	150 mg/kg	Piglets’ pre-starter (28–47 day of life) and starter (48–74 day of life)	The low level of ZnONPs (150 mg/kg) can exhibit a similar antidiarrheal action as high therapeutic doses of ZnO (from 1000 to 4000 mg/kg)	Szuba-Trznadel et al. (2021)
ZnONPs	20, 40, or 60 mg/kg	Broiler chickens (Ross 308)	Improved broiler chicken growth, nutritional digestibility, carcass criteria, and liver and kidney functions under heated climatic circumstances for all ZnONPs doses	Abdel-Wareth et al. (2022)
Nano-Ag	4, 8 and 12 mg/L of drinking water	Broiler chickens	Negative effect of nano-Ag a on body weight, feed intake, feed conversion rate when compared with the control group	Ahmadi and Rahimi (2011)
AgNPs	0.5 and 1.0 mg AgNPs/kg body weight	Rabbits	Reduced total cholesterol and triglycerides in plasma when compared to the control group; the highest concentration of GPX and MDA for AgNPs	Abdelsalam et al. (2019)
CuNPs	0.3 mL of 50 mg/L of drinking water	Broiler chickens *in ovo*	A positive effect on broiler chickens’ performance (e.g., body weight) as compared to the control group	Mroczek-Sosnowska et al. (2015b)
CuNPs	10 mg/L of drinking water (< 100 nm)	Broiler chickens	Improved immunity, behavior and growth performance more efficiently than CuSO_4_	El-kazaz and Hafez (2020)
CuNPs	50 mg/kg (2–15 nm)	Chicken embryo injection (1st day of incubation)	Improved metabolic rate and no harmful effect on embryo development	Scott et al. (2016)
Nano-Cu	5, 10 and 15 mg/L of drinking water (5 nm)	Broiler chickens (1–7 weeks old)	Increased content of Cu in the blood; decreased absorption of Zn and Ca; No effect on Fe absorption	Ognik et al. (2016b)
Nano-Cu	50 mg/kg (15–70 nm)	Chicken embryo (1st day of incubation)	Positive effect on chicken growth performance and improved percentage of breast and leg muscles	Mroczek-Sosnowska et al. (2015a)
CuNPs	50 mg/kg	Broiler chickens injection (1st day of incubation)	Increased accumulation of Cu in the liver and spleen organs	Mroczek-Sosnowska et al. (2014)
Nano-Cu	100 mg/kg	Broiler chickens (1–32 days old)	No effect on the growth performance and digestibility of nutrients	Sarvestani et al. (2016)
Nano-Cu	50 mg/kg (37.3 nm)	Chicken embryo injection (1st day of incubation)	Pro-angiogenic properties at a systemic level to a greater degree than inorganic form of copper (CuSO_4_)	Mroczek-Sosnowska et al. (2015b)
AuNPs	5 and 15 mg/L of drinking water	Cobb broiler chickens	A positive effect of lower NPs dose on growth performance without any significant difference in immunological parameters and oxidative stress damage in organs (spleen, liver, bursa of fabricius, thymus) as compared to control	Hassanen et al. (2020)
Nano-Se	0.3 mg/kg (50–100 nm)	Laying hens (9–20 weeks old)	Increase immunization cutaneous basophil hypersensitivity (CBH) response	Mohapatra et al. (2014a)
SeNPs	0.075, 0.1125. 0.1875 and 0.225 mg/kg (30–60 nm)	Broiler chickens (1–35 days old)	Improved SOD and GSH-Px activity in the serum; improved the oxidation resistance; decreased MDA level	Aparna and Karunakaran (2016)
Nano-Se	0.075, 0.1125, 0.1875 and 0.225 mg/kg (30–60 nm)	Broiler chickens (1–5 weeks old)	Improved the oxidation resistance with Nano-Se supplementation; increased the expression of liver GSHP × 1 mRNA gene	Aparna et al. (2017)
Nano-Se	0.5 mg/kg (20–80 nm)	Broiler chickens (1–7 weeks old)	Improved immunity and total antioxidant activity of serum with 0.5 mg/kg nano-Se	Bagheri et al. (2015)
Nano-Se	0.15 and 0.30 mg/kg (feed or drinking water) (80 nm)	Broiler chickens (400 days old)	Significant improvement of growth performance and Se content in liver and thigh tissues with increasing nano-Se dose	Selim et al. (2015a)
Nano-Se	0.15 and 0.30 mg/kg (80 nm)	Broiler chickens (1–40 days old)	Better growth rate, feed efficiency and meat quality	Selim et al. (2015b)
Nano-Se	0.1, 0.2, 0.3, 0.4 and 0.5 mg/kg	Broiler chickens (1–7 weeks old)	Improved growth performance, carcass parts and immunity; improved anti-ND hemagglutination inhibition titer	Ahmadi et al. (2018)
Nano-Se	0.15, 0.30, 0.60 and 1.20 mg/kg	Broiler chickens (1 day old)	Improved growth performance, a plateau for gain/feed and survival ratio for 0.15–1.20 mg/kg of nano-Se, increase with nano-Se dose increase; increase in Se concentrations in serum, liver and breast muscle (higher for Nano-Se than for sodium selenite); increased serum GSH-Px activity	Hu et al. (2012)
Nano-Se	50, 150 and 300 ppb	Giriraja chickens (1–56 days old)	Increased water holding capacity of meat; no impact on carcass characteristics and production parameters	Prasoon et al. (2018)
Nano-Se	0.10 mg/kg	Lambs	Reduced the oxidative stress; enhanced the activity of blood glutathione peroxidase; increased the lambs weight gain; no effects on Cu, Zn and Fe levels in blood	Yaghmaie et al. (2017)
Nano-Se	0.3 mg/kg	Goats	Improved growth performance (finial body weight (BW) and average daily gain); improved serum oxidant status (GSH-Px, SOD, CAT activity); increase in Se concentration in blood and tissues for nano-Se as compared to sodium selenite	Shi et al. (2011b)
Nano-Zn, Nano-Cu, Nano-Se	4, 8, 12 and 16 µg/egg (< 100 nm)	Broiler chickens *in ovo* injection (18^th^ day of incubation)	No harmful effects on the developing embryo and hatchability percentage	Joshua et al. (2016)
Calcium phosphate NPs (CPNPs)	50, 60, 70, 80, 90 and 100% (20–90 nm)	Broiler chickens (1–28 days old)	Increase in cumulative feed intake for NPs groups as compared with dicalcium phosphate (100%); higher body weight gain for 50 and 60% of NPs; best feed conversion ratio for 50% NPs	Vijayakumar and Balakrishnan (2014)
Calcium phosphate NPs (CPNPs)	25, 50, 75 and 100% of CPNPs (51-200 nm) with or without dicalcium phosphate (DCP)	Broiler chicks	CPNPs with 50% level increased body weight gain without altering feed conversion ratio, biochemical parameters, and carcass characteristics similarly to the 100% DCP	Samanta et al. (2019)
Nano-Cr	200 and 400 µg/kg	Babcock layer chickens	No effect on growth performance, egg production and egg weight; except Cu, significant increase in the retention of Cr, Zn, Fe, Ca and P; nano-Cr (400 µg/kg) increased the concentration of minerals in some organs such as in plasma (Cr and Zn), liver and egg shell (Cr, Ca and Zn), and Zn in egg yolk	Sathyabama and Jagadeeswaran (2016)

Metal/metal oxide nanoparticles supplemented to animal diet can improve not only animal performance and productivity such as meat, milk, and eggs production, but also ameliorate the quality of the animal-derived products (Hill and Li 2017; Mekonnen 2021). Usually, enrichment of animal products like meat or eggs with microelements is observed (e.g., Shi et al. 2011b; Hu et al. 2012; Mroczek-Sosnowska et al. 2014; Selim et al. 2015a; Sathyabama and Jagadeeswaran 2016; Eskandani et al. 2021). Nanoparticles can increase the bioavailability of nutrients to animals due to their nanoscale. Absorption of nutrients in the gut may be enhanced and can exert strengthened biological effects in the target tissues of animals (Bunglavan et al. 2014; Vijayakumar and Balakrishnan 2014; Hill and Li 2017; Youssef et al. 2019; Amlan and Lalhriatpuii 2020). Minerals in the nanoparticle form can pass through the stomach wall and into body cells faster than common inorganic salts with a larger particle size (Bunglavan et al. 2014). Hu et al. (2012) showed that in the case of selenium, its nano-form transfer from the intestinal lumen to the body was significantly higher than for sodium selenite with significantly lower intestinal retention of nano-Se than for the inorganic form. Retention of nano-Se in the body was more efficient than sodium selenite. Metal-containing nanoparticles can be a solution for nutrient deficiencies in animals and can enrich animal products with microelements. Additionally, minerals in the form of nanoparticles are believed to reduce intestinal minerals antagonism and thus minimize their excretion and environmental pollution (Gopi et al. 2017).

Supplementation of nano-minerals can also reduce oxidative stress and improve serum oxidant status. The increase in activity of antioxidant enzymes such as glutathione peroxidase (GSH-Px), superoxidase dismutase (SOD), catalase (CAT), and total antioxidant activity (AOA) while decrease in malondialdehyde (MDA) is a common phenomenon, for example for SeNPs (Shi et al. 2011b; Hu et al. 2012; Aparna and Karunakaran 2016; Yaghmaie et al. 2017), ZnNPs (Ahmadi et al. 2014; Ibrahim et al. 2017; El-Katcha et al. 2018; El-Maddawy et al. 2022) and AgNPs (Abdelsalam et al. 2019). Antioxidant enzymes are responsible for elimination of reactive oxygen species, whereas MDA serves as an index of antioxidant status, is the end product of the oxidative stress (Shi et al. 2011b). Shokraneh et al. (2020) showed that *in ovo* injection of nano-selenium (40 μg) and nano-zinc (500 μg) alleviated the negative effects of heat stress, induced by high eggshell temperature during late incubation, through boosting antioxidant activity and reducing oxidative stress (increased GSH-Px and SOD activity, while decreased corticosterone, cortisol, T4 and T3) in broiler hatchlings.

Metal/metal oxide nanoparticles are also known to be a potential alternative to antibiotics (Hill and Li 2017; Swain and Prusty 2021a). Examples of the antimicrobial effects of nano-minerals on different animal species are presented in Table 2. Zinc, silver, and selenium nanoparticles are mainly examined as antimicrobial agent. The best known are silver nanoparticles, which exhibit a broad spectrum of antimicrobial properties, for example against *Escherichia coli*, *Staphylococcus aureus*, *Salmonella* *typhimurium*, *Salmonella pullorum, Klebsiella*, *Pseudomonas*, and yeast (Kim et al. 2007; Wahab et al. 2010; Mekonnen 2021; Li et al. 2021), in addition to fungi (*Aspergillus flavus*, *A. niger* and *A. ochraceus*) and produced mycotoxins (Hassan et al. 2013; Swain and Prusty 2021a), as well as *Eimeria* species in rabbits and chickens (Chauke and Siebrits 2012; Abd El Megid et al. 2018; El-Maddawy et al. 2022). Generation of the free radicals or reactive oxygen species by Ag nanoparticles can inhibit microbial growth as their interaction with bacterial cells may lead to breaking down the bacterial cell wall (Kim et al. 2007).

**Table 2.** Ameliorative and antimicrobial effects of nano-minerals on different animal species – *in vitro* and *in vivo* studies.

Type of NPs	Dose/size of NPs	Animal species/age	Pathogen/stress	Effects	References
ZnONPs	10, 20, 30 and 40 mg/kg	Broiler chickens	Multidrug resistant *Staphylococcus aureus*-induced footpad dermatitis	ZnONPs prevented multidrug resistant *S. aureus*-induced footpad dermatitis and ameliorated the negative effects on behavior (standing and walking) and performance	Mahmoud et al. (2021)
ZnNPs	45, 90, 135 and 180 mg/kg	Broiler chickens	*Escherichia coli* and *Salmonella* sp.	Reduction in population of pathogenic intestinal bacteria	Hidayat et al. (2021)
ZnONPs	20 mg/kg	Broiler chickens	Mixed *Eimeria* species (*E. maxima*, *E. mivati*, *E. acervulina*, and *E. tenella*)	In the infected group, enhanced growth performance, decreased average oocyst count numerically, significantly lowered gut lesion score; ZnONPs enhanced PCV and Hb percent, and RBC count considerably, boosted plasma carotenoids levels and antioxidant activity, and lowered MDA	El-Maddawy et al. (2022)
ZnO quantum dots (ZnO QDs)	(3-6 nm)	Chicks	*Salmonella pullorum*	ZnO QDs effectively reduced the mortality of infected chickens by regulating intestinal flora balance, preserving the liver and gut, and altering the balance of antioxidation systems	Li et al. (2021)
ZnO QDs	(3-6 nm)	*In vitro*	*E. coli*, *S. aureus* and *S. pullorum*	The growth of all microorganisms was suppressed by ZnO QDs at a rate of 87.1, 94.7, and 85.6%, respectively with the lowest inhibitory concentrations of 0.781, 0.0976, and 0.195 mg/mL	Li et al. (2021)
Nano-Ag	25 mg/L	Pigs	African swine fever virus	Reduced microbial contamination in the pig house	Dung et al. (2020)
AgNPs	150 µg/bird, drinking water (15 nm)	Broiler chickens	*C. perfringens* induced necrotic enteritis	AgNPs reduced the severity of clinical signs, mortality rate, pathological lesions in the intestine and liver of infected birds; reduced *C. perfringens* colonization in the small and large intestine	Salem et al. (2021)
AgNPs	50 mg/L of drinking water (15 nm)	Broiler chickens	Pathogenic *Escherichia coli*	Reduced mortality rate from 14.1% in control to 4.92% in group treated with AgNPs	Kumar and Bhattacharya (2019)
AgNPs	15 mg/L of drinking water	Chickens	*Eimeria tenella*	AgNPs induced a slightly better cecal lesion score than the untreated birds; 50% less oocysts in the feces compared to the untreated group; no effect on the weight gain	Chauke and Siebrits (2012)
AgNPs	50 mg/L	Broiler chickens	*Campylobacter jejuni*	AgNPs had no antibacterial effect on *C. jejuni* and different intestinal bacteria; impaired broiler growth and immune functions	Vadalasetty et al. (2018)
Nano-Se	Not available	Broiler chickens	Mild *Clostridium perfringens* challenge model	Upregulated gene expression of gut barrier function; promoted shifts in gut bacterial enzyme activity to increase energy uptake in challenged birds and increased collagenase activity	Konieczka et al. (2020)
Nano-Se	0.4 g/kg	Quail	Gut microbiota	Decreased total bacterial count, total yeast and molds count, *Coliform*, *Escherichia coli*, *Enterococcus* spp., and *Salmonella* spp. colonization, increased lactic acid bacteria counts than those in the control group	Alagawany et al. (2021)
Nano-Se	100, 200, 300, 400 and 500 mg/mL (65.2 nm)	*In vitro* (Disc diffusion method)	Bacterial isolates (*Bacillus cereus*, *S. aureus*, *Listeria monocytogenes*, *Salmonella Typhi*, *E. coli*, *Klebsiella pneumonia*); fungal isolates (*Candida tropicalis*, *C. albicans*, *C. glabrata*, *Aspergillus niger*, *A. flavus*, *A. fumigates*)	SeNPs exhibited an antimicrobial activity against all tested bacterial isolates with more resistance showed Gram-negative bacteria; inhibition of all tested fungi isolates; antioxidant activity of SeNPs (showed by scavenging ABTS and DPPH radicals)	Abdel-Moneim et al. (2022)
SeNPs	5 mM and 10 mM; (79.4- 44.3 nm)	*In vitro* (Disc diffusion method)	*Klebsiella pneumonia*, *Salmonella abony*, *E. coli*, *Candida albicans*	Antimicrobial activities against all tested microorganisms with superior effect on *S. abony,* followed by *K. pneumoniae*, then *C. albicans* and *E. coli*	Abbas et al. (2021)
SeNPs	0.5 mg/kg	Laying hens	Deoxynivalenol (DON) toxicity	SeNPs provided effective anti-oxidative protection against DON toxicity; reduced DON’s effect on egg production rate, egg quality and serum calcium level	Qu et al. (2017)
Gold, Silver, Copper NPs	25 and 50 mg/kg	*In vitro*	*E. coli*, *Streptococcus uberis*, *S. aureus*, *C. albicans* and *C. krusei* isolated from clinical cases of bovine mastitis	AgNPs (both doses) and copper (50 mg/kg) showed the highest inhibiting activity against the pathogens while gold NPs were the weakest	Wernicki et al. (2014)

## 4. Metal nanoparticles in the nutrition of particular animal species

### 4.1. Zinc (Zn) nanoparticles

The significance of zinc in animal feeding, its bioavailability (absorption) in the body, properties of Zn nanoparticles, their effect on biological systems/animal performance (growth, milk production), rumen fermentation, immunity, reproduction, antibacterial activity, and toxicity were described in detail in the reviews of Swain et al. (2016), Bąkowski et al. (2018), Yusof et al. (2019), Amlan and Lalhriatpuii (2020), and Abdelnour et al. (2021). Zinc oxide nanoparticles (ZnONPs) have good prospects for the use as one of the new antibiotics (Yusof et al. 2019; Ouyang et al. 2021) and anticoccidial alternatives (El-Maddawy et al. 2022). The nano-zinc can also increase the activity of the superoxide dismutase enzyme, which constitutes a very important antioxidant defense against oxidative stress (Gonzales-Eguia et al. 2009; Refaie et al. 2015; Hidayat et al. 2021). Examples of zinc nanoparticles applications in various species of animal's nutrition are presented below.

#### 4.1.1. Poultry

Eskandani et al. (2021) compared the effect of different Zn sources (control – ZnSO_4_, organic – amino acid complex and Zn NPs at doses 30, 50, 70 and 90 mg/kg of diet) in the diet of broiler chickens on performance, carcass characteristics, humoral immunity, meat quality and Zn content in meat. Improvement of these parameters was observed for ZnNPs applied at a dose of 70 mg/kg of diet. Hidayat et al. (2021) tested several doses of ZnNPs (45, 90, 135 and 180 mg/kg) on broilers. The addition of zinc nanoparticles up to 90 mg/kg had a positive effect on performance, antioxidant activity, and reduction in population of pathogenic intestinal bacteria (*E. coli* and *Salmonella* sp.). In addition, they exerted antifungal activity against toxigenic moulds in feed systems (*Aspergillus flavus*, *A. ochraceus* and *A. niger*) (Hassan et al. 2013). The weight of lymphoid organs in broiler chickens increased as well as humoral immunity was improved by supplementation of nano-Zn (0.06 mg/kg diet) similarly to that of 15 mg/kg diet of organic Zn (Sahoo et al. 2014). Hafez et al. (2020) reported an enhancement of IgY production and cellular immunity (total lymphocyte count, macrophages, phagocytic activity, and index) in broiler chickens by ZnONPs supplementation compared to ZnO. Nano-form of zinc (20, 40, 60 and 80 μg/egg), *in ovo* supplemented in fertile broiler eggs, had no harmful effect on embryo development, did not influence the hatchability and can be used to improve the post-hatch performance of broilers (Joshua et al. 2016).

Supplementation of zinc in nano-form into the diet of turkey hens in the dose covering 10% of the demand (10 mg/kg of diet) maintained homeostasis in turkey muscles, as indicated by the activity of the aminopeptidases (alanyl, leucyl, and arginyl) (Jóźwik et al. 2018).

Abbasi et al. (2017) evaluated dose–response of ZnONPs supplementation (25, 50, 75 and 100 mg/kg of diet) to Japanese quails from 10 to 40 days of age. Growth performance was increased significantly in zinc supplemented birds between 20 to 30 days. The optimal ZnONPs levels for body weight gain of Japanese quails were 90, 70 and 59 mg/kg of diet for birds 10–20 days old, 20–30 days old, and 30–40 days old, respectively. The relative weights of testes and thigh were increased significantly by increasing dietary Zn levels. Among doses of biological nano-zinc (0.1, 0.2, 0.3 and 0.4 g/kg diet) administered to growing Japanese quails, ZnNPs at doses of 0.1–0.3 g/kg diet resulted in an increase in the activity of ALT, AST, LDH, superoxide dismutase, glutathione peroxidase, and concentrations of malondialdehyde, and immunoglobulins G and M (IgG and IgM), as well as an increase in beneficial microbial populations. The total cholesterol, high-density lipoprotein, and low-density lipoprotein concentrations are the only among liver profile parameters influenced by ZnNPs. A significant improvement in body weight, weight gain, feed intake, and feed conversion ratio were observed by 0.2 g/kg of ZnNPs supplementation (Reda et al. 2021). Nano- or micro-particles of ZnO were supplied to Japanese quails from 47 to 75 days of age with doses of 49, 74, 99, and 124 mg zinc per kg/diet to investigate their effects on performance, fertility, hatchability, and egg quality characteristics (Abbasi et al. 2022). Quails fed diets supplement with ZnO showed significant improvement of the eggshell thickness regardless of particle size, as well as higher egg weight and eggshell surface as compared with ZnO-non-supplemented birds. The maximum egg production percentage was achieved when 67 or 72 mg/kg of dietary zinc was supplied from nano- and micro-ZnO, respectively, in addition to a considerable enhancement of fertility, which was shown by nano-ZnO supplementation. Hence, nano-ZnO could decrease the zinc requirement in laying Japanese quail (Abbasi et al. 2022).

Zinc in the diet was shown to be effective in maintaining laying bird performance, whereas Zn-glycine was found to be responsible for superior eggshell quality (Olgun and Yildiz 2017). However, in comparison to the Zn-sulphate and Zn-glycine forms, supplementing feed of laying hens with zinc nanoparticles (50, 75, and 100 mg/kg of diet) had a negative effect on eggshell thickness and bone mechanical characteristics while increasing egg weight (Olgun and Yildiz 2017). Despite this, Abedini et al. (2018) found that laying hens fed 40 and 80 mg Zn/kg of feed as ZnONPs had improved eggshell thickness and strength, as well as egg mass. Even, dietary Zn source either inorganic, organic, or nano-Zn had no effect on egg mass and eggshell quality (Cufadar et al. 2020). Moreover, nano-Zn supplementation may aid in egg quality preservation through increasing of Zn content in egg yolk (Alagawany et al. 2018).

Dietary ZnONPs have shown to be beneficial in ameliorating the negative effects of heat stress (Ramiah et al. 2019; Shokraneh et al. 2020). Akhavan-Salamat and Ghasemi (2019) compared three sources of dietary Zn (Zn oxide, Zn-Methionine (Zn-Met), and Zn oxide nanoparticles – ZnONPs) and three doses of supplemental Zn (low: 20 mg/kg, adequate: 40 mg/kg, and high: 80 mg/kg of diet) to broiler chickens reared under heat stress beginning from 14 to 42 days. Dietary Zn had no influence on growth performance induced by Zn content, but total and IgM antibody titers against sheep red blood cells (SRBC) were increased significantly by high-Zn level. The average daily weight gain from 0 to 42 days, relative spleen weight, and serum superoxide dismutase activity in the Zn-Met and ZnONP supplemented groups were significantly greater than those of Zn oxide treatment. Moreover, overall FCR and serum malondialdehyde concentration were decreased by dietary supplementation of Zn-Met and ZnONPs. They concluded that the supplementation of Zn in the forms of Zn-Met and ZnONPs at a dose of 40 mg/kg of diet could beneficially affect the growth performance, immune response, and antioxidant status of broiler chickens reared under high temperatures.

Moreover, zinc nanoparticles have shown antibacterial activity. Mahmoud et al. (2021) indicated that dietary supplementation of 10, 20, 30, or 40 mg/kg of diet by ZnONPs prevented multidrug resistant *Staphylococcus aureus*-induced footpad dermatitis and ameliorated the negative effects on behavior (standing and walking), performance (daily body weight gain, feed conversion ratio) and welfare changes in broilers. In addition to the significantly higher latency to lie time (LLT), lower serum cortisol concentration was observed only for groups with ZnONPs doses − 30 and 40 mg/kg of diet and non-infected groups in comparison with the *S. aureus* group. Additionally, zinc oxide nanoparticles showed an anticoccidial effect in broiler chickens experimentally infected with a mixture of *Eimeria maxima*, *E. acervulina*, *E. mivati*, and *E. tenella* and appeared as effective as diclazuril, a chemical anticoccidial drug, through evaluation of growth performance, parasitological and hematological parameters, as well as antioxidant activity (El-Maddawy et al. 2022).

#### 4.1.2. Pigs

Ouyang et al. (2021) showed that porous nanoparticles of ZnO (500 and 1000 mg/kg of diet) had better anti-diarrhoea and growth promoting effect when compared with ordinary-size ZnO (3000 mg/kg of diet). There were no significant differences in average daily feed intake or average daily gain of piglets among the tested groups. Supplementation of ZnO nanoparticles to the diet of weaned piglets (500, 1000 and 2000 mg/kg of diet) significantly increased their weight gain when compared with inorganic ZnO. Feces of piglets fed with ZnONPs contained lower number of the total aerobic bacteria and coliform counts than ZnO groups (Kociova et al. 2020). The application of Zn nanoparticles in pig's diet can be one of the strategies for preventing diarrhea, which is common in weaned piglets. Szuba-Trznadel et al. (2021) showed that low levels of ZnO nanoparticles (150 mg/kg of diet) can exhibit a similar antidiarrheal action as high therapeutic doses of ZnO (from 1000 to 4000 mg/kg of diet). Additionally, average daily gain in the group with ZnONPs was significantly higher than in the group with inorganic zinc supplied as ZnSO_4_. In the work of Cui et al. (2021), nano-zinc (200, 300, 400, 500 mg/kg of diet) had a positive effect on piglets – especially 500 mg/kg of diet – improved intestinal antioxidant capacity (increase in the superoxide dismutase activity, total antioxidant capacity of duodenum and ileum, catalase in the jejunum and ileum, copper–zinc superoxide dismutase and glutathione peroxidase activities in the ileum and reduction in the malondialdehyde content in the jejunum), enhanced intestinal immunity (immunoglobulins concentration), improved intestinal morphology of duodenum, jejunum and ileum and reduced zinc content in liver and feces. But zinc nanoparticles had no significant effect on growth performance (Cui et al. 2021).

#### 4.1.3. Ruminants

The effect of nano-zinc on growth performance, feed digestibility, milk yield parameters, antioxidant status and health, serum antioxidant parameters, immune response and serum/milk composition and reproduction of ruminants was described in detail by Abdelnour et al. (2021). Many authors showed the promising positive effects of zinc nanoparticles on animal performance (Cui et al. 2021; Eskandani et al. 2021). In the research of Abdollahi et al. (2020), pre- and post-weaning calves obtained in the diet zinc in the common form – ZnO and as high-surface ZnO nanoparticles. Calves receiving ZnONPs showed higher level of the post-weaning dry matter intake, nutrient digestibility, blood hematocrit and blood Zn concentration than calves supplemented with common ZnO. Additionally, both Zn forms positively affected the concentration of rumen volatile fatty acids, decreased rumen ammonia–N concentration, increased the blood total antioxidant capacity, and lowered the incidence of pneumonia and diarrhea in calves (Abdollahi et al. 2020). Bakhshizadeh et al. (2019) compared the effect of three different zinc sources (control diet without zinc supplementation, zinc oxide, zinc glycine and nano-zinc) at a dose of 60 mg/kg of diet on dairy cows. There were no differences between examined groups in dry matter intake, milk yield, body weight, biochemical and hematological parameters (with exception of blood urea nitrogen concentration, which was lower in the zinc glycine and nano-zinc group than in the zinc oxide and the control group). Zinc supplementation in the form of zinc nanoparticles increased the superoxide dismutase activity and plasma Zn concentration when compared with ZnO and control group. As such, nano- and organic Zn sources in the diet of dairy cows were more suitable feed additives than inorganic Zn.

Zinc oxide nanoparticles were also proposed to control aflatoxin B1 contamination caused by fungi belonging to the genera of *Aspergillus* (*A*. *flavus*, *A*. *ochraceus*, and *A*. *niger*) in cattle feed – yellow corn (Hassan et al. 2017). Among tested concentrations − 0.025, 0.05, 0.1, 0.2, 0.3 and 0.4 mg/kg of feed, degradation of aflatoxin required high doses of ZnONPs − 0.3 mg/kg of feed. However, frequent addition of nanoparticles to feeding may result in toxicity to animals, therefore in this research the combination of ZnONPs (0.05 mg/kg of feed) and ozone fumigation for 10 minutes was applied to achieve the same degradation effect.

Hozyen et al. (2019) prepared two forms of synthesized ZnONPs (sonochemically synthesized capped ZnONPs were compared with aggregated uncapped ZnONPs) and tested them at different concentrations for their antimicrobial effects *in vitro* against *Staphylococcus aureus*, *Escherichia coli* and *Klebsiella pneumoniae* isolated from milk samples of affected cows with clinical mastitis. Capped dispersed ZnONPs showed higher antibacterial activity against *S*. *aureus*, *E*. *coli*, and *K*. *pneumoniae* than aggregated uncapped ZnONPs at same concentrations. They observed that the inhibition zone for two forms of ZnONPs was concentration dependent. Additionally, Gram-positive *S. aureus* showed higher resistance to both ZnONPs than Gram-negative *E*. *coli* and *K*. *pneumoniae*.

Swain et al. (2021b) evaluated the effects of nano-zinc (ZnNPs, dose 25 or 50 mg/kg diet) and inorganic zinc (dose of 50 mg/kg diet) on immune status, and hormone profiles (T_3_, T_4_ and Insulin-like growth factor 1 (IGF-1) in goats through 90 days feeding trial. ZnNPs supplementation by 25 mg/kg of diet had a similar improved effect on immunity and IGF-1 serum level compared with inorganic Zn at 50 mg/kg dose without changes of thyroid hormones profile, while 50 mg of ZnNPs/kg diet showed the highest humoral and cell-mediated immunity.

### 4.2. Silver (Ag) nanoparticles

The effect of silver nanoparticles on animal productive performance, blood parameters, immune system, their accumulation in animals’ organs and intestinal microbiome was summarized in the reviews of Fondevila (2010), Gangadoo et al. (2016), Bąkowski et al. (2018), etc. Since silver compounds are known for their antimicrobial properties, silver nanoparticles are considered as a potential antimicrobial feed additive (Grodzik and Sawosz 2006; Sawosz et al. 2007; Pineda et al. 2012; Ognik et al. 2016a; Kumar and Bhattacharya 2019; Youssef et al. 2019; Awaad et al. 2021; Niemiec et al. 2021). Silver nanoparticles show inhibitory effects on various species of bacteria, including *Escherichia coli* and *Staphylococcus aureus* (Sawosz et al. 2007; Pineda et al. 2012; Kumar and Bhattacharya 2019), *Salmonella, Streptococcus*, and total mesophilic bacteria (Dobrzanski et al. 2010) and can be used in a variety of animal species.

#### 4.2.1. Poultry

Most of the experiments concerning AgNPs testing in poultry are related to their antimicrobial properties and effect on the microbial profile. In the work of Awaad et al. (2021), 1-day old broiler chickens infected with *Escherichia coli* O78 were fed with the diet supplemented with AgNPs at doses 4, 6 and 8 mg/kg of diet. The treatment of colisepticemic chickens with the lowest dose improved productive performance (weekly individual body weight, feed intake and feed conversion ratio), reduced gross and histopathological lesion scores and virulence genes expression (Awaad et al. 2021). Ognik et al. (2016a) showed that silver nanoparticles hydrocolloid and lipid-coated nanosilver hydrocolloids at a dose of 5 mg per kg body weight per day, tested on broiler chickens increased the total number of aerobic mesophilic bacteria and decreased the number of Coli group bacteria. Supplementation of silver nanoparticle had no effect on growth performance and jejunum morphology. Kumar and Bhattacharya (2019) added silver nanoparticles to drinking water (50 mg/L) of chickens. AgNPs revealed antibacterial activity against *Escherichia coli*, significantly reduced the poultry mortality rate, increased significantly feed intake and body weight but had no significant effect on feed conversion ratio. Kout-Elkloub et al. (2015) indicated that broilers fed with diet supplemented with AgNPs had decreased number of *E. coli* as compared to the control and had no effect on microflora represented by *Lactobacillus*. Among tested doses of AgNPs (2, 4, 6, 8 and 10 mg/kg of diet) in the diet of broilers, the dose of 4 mg/kg of diet caused the greatest body weight gain, the highest final body weight and the best feed conversion ratio among the tested groups. Level of nano-silver had no significant impact on overall feed intake. AgNPs decreased serum total lipids and significantly increased total serum antioxidant capacity in all treatments. Ahmadi and Kurdestany (2010) supplemented silver nanoparticles to the drinking water of male broiler chickens at doses 5, 15 and 25 mg/L. This additive had no effect on performance. Weight of bursa was reduced when compared with the control group and the highest decrease was observed for the highest AnNPs dose. Nano-silver increased catalase and glutathione peroxidase activity and malondialdehyde content and decreased superoxidase dismutase activity. Silver nanoparticles added to drinking water of Japanese quails significantly increased the number of bacteria (*Lactobacillus* spp., *Leuconostoc lactis*, *Actinomyces naeslundii*), had no effect on *Enterococcus faecium* population, the number of *E. coli* and other Enterobacteriaceae. Additionally, AgNPs had no negative effect on enterocytes of duodenal villi (Sawosz et al. 2007). Salem et al. (2021) studied the antimicrobial activity of silver nanoparticles in broiler chickens experimentally infected with *C. perfringens* associated necrotic enteritis (NE). 30 µg/bird of AgNPs was administered to chickens for 5 successive days post infection via crop gavage. AgNPs reduced the severity of clinical signs, mortalities, and pathological lesions in the intestine and liver as well as *C. perfringens*' colonization in the intestine and ceca in infected birds. However, their residues were detected in the birds’ muscles. Grodzik and Sawosz (2006) showed that silver nanoparticles, injected into fertilized chicken eggs, did not influence the development of chicken embryos, but decreased the number and size of lymph follicles in the bursa of Fabricius. Further investigation on the application of nano-silver in the commercial poultry production, could lead to the development of feeding strategies for chickens aiming at reducing the use of antibiotics as growth promoters (Pineda et al. 2012).

Nevertheless, literature data also indicate the unfavorable effects of supplementing the diet of poultry with silver nanoparticles. Ahmadi and Rahimi (2011) tested colloidal nano-silver added to drinking water of broilers at doses of 4, 8 and 12 mg/L. This nano-element had a negative effect on body weight, feed intake and feed conversion rate when compared with the control group. AgNPs decreased growth performance and economic traits in broilers. Similar negative effect was obtained by 50 mg/L in drinking water for broiler chickens in addition to impaired immune functions (Vadalasetty et al. 2018). 15 mg of AgNPs/L did not show either a significant growth promoting effect or conclusive coccidiostatic activity (Chauke and Siebrits 2012). Moreover, nano-silver accumulated in breast muscle, liver, femur and feces. In the work of Pineda et al. (2012), silver nanoparticles were injected into fertile eggs, as well as added to drinking water during the post-hatch period. Such treatment reduced feed intake and body weight, decreased lactose-negative enterobacteria and lactic acid bacteria in the cecum but had no effect on the bacterial populations in the ileum and concentrations of IgG and IgM in plasma.

#### 4.2.2. Pigs

Silver nanoparticles, supplied at doses 20 and 40 mg/kg of weanling pigs feed, increased linearly their daily growth with the increasing dose of metallic Ag, and reduced ileal concentration of coliforms, whereas lactobacilli remained unaffected. Feed intake was higher for a lower dose of AgNPs (Fondevila et al. 2009). Interesting properties of nano-silver were demonstrated in the study of Dung et al. (2020). Silver nanoparticles exhibited antiviral ability against African swine fever virus, which is the cause of a highly contagious and fatal disease in domestic swine. Nano-silver at a dose of 25 mg/L significantly reduced the microbial contamination in the pig house and constitutes a promising disinfectant.

#### 4.2.3. Sheep and goat

Farouk et al. (2020) investigated *in vitro* antibacterial activity of silver nanoparticles against multidrug-resistant (MDR) *Salmonella* isolates recovered from diarrhoeic sheep and goats as well as assessed their *in vivo* treatment efficacy in mice against *S. enteritidis*. 4% and 3.6% of sheep and goats, respectively, were infected with MDR *Salmonella* isolates and all of these isolates were affected efficiently with AgNPS with MIC (minimum inhibitory concentration) of ≤0.02–0.313 μg/mL and MBC (minimum bactericidal concentration) of 0.078–1.250 μg/mL. *In vivo*, AgNPs reduced the number of viable *S. enteritidis* in feces of infected mice, that completely stopped between the 4th and 6th day of treatment, as well as AgNPs suppressed inflammatory reaction caused by *S. enteritidis*.

#### 4.2.4. Rabbits

Abdelsalam et al. (2019) tested subcutaneous injections of silver nanoparticles (0.5 and 1.0 mg AgNPs/kg body weight) twice a week for four consecutive weeks. Administered AgNPs had no significant effect on feed intake, feed conversion ratio and average daily gain. Rabbits supplemented with 0.5 mg of AgNPs showed lower concentration of total cholesterol and triglycerides in plasma than the control group, the lowest total antioxidant capacity and the highest concentration of malondialdehyde and glutathione peroxidase activity. Additionally, the accumulation of silver in blood plasma and meat increased with the increasing dose of AgNPs.

### 4.3. Copper (Cu) nanoparticles

Cu is an essential trace element in animals and an essential cofactor for several cellular enzymes. It can promote participation in hematopoiesis, bone formation, enhancement of immunity, increase in resistance to foreign pathogens and antioxidant capacity (Gangadoo et al. 2016). Furthermore, copper is involved in various physiological and biochemical processes (Gangadoo et al. 2016; Scott et al. 2018). The importance of Cu in the animal diet, physicochemical and biological properties of CuNPs (nutritional and physiological characteristics, antibacterial activity, immunological and toxicological effects) were described in reviews, for example by Scott et al. (2018), Amlan and Lalhriatpuii (2020). Copper nanoparticles supplemented to animal feed act as a growth promoter, immune stimulant, and antibacterial and antifungal agent (Morsy et al. 2021).

#### 4.3.1. Poultry

Copper nanoparticles are being studied in many aspects in poultry nutrition. Aminullah et al. (2021) compared the effect of different forms of copper supplemented to the diet of Swarnadhara breeder hens. It was found that the commonly used inorganic form of Cu (CuSO_4_) can be replaced with 50% of organic (Cu proteinate) or 25% of nano-form without negative impact on hens’ productivity, hatchability, progeny, and egg quality. Miroshnikov et al. (2015) administered copper nanoparticles to broiler chickens by single intramuscular injection. Quick growth stimulation and metabolic changes were observed. Copper and protein concentration in serum was changed only 7 and 21 days after injection. Ognik et al. (2016b) evaluated the effect of copper nanoparticles supplementation on the intestinal absorption of calcium, iron and zinc. Oral administration of copper nanoparticles (5, 10 and 15 mg/L) to Ross 308 chickens led to the accumulation of this element in the intestinal walls. For a dose of 15 mg/L of nano-copper, the concentration of Cu increased in plasma. It is supposed that copper accumulated in intestines can reduce calcium and zinc absorption but has no effect on iron absorption.

For poultry, *in ovo* application of copper nanoparticles has shown promising results. Mroczek-Sosnowska et al. (2015b) demonstrated that *in ovo* administration of copper nanoparticles (0.3 mL of 50 mg/L) had a positive effect on broiler chickens’ performance (e.g., body weight) as compared to the control group. Group with nano-copper had significantly lower feed conversion rate and mortality and higher percentage of breast and leg muscles in the carcass when compared with the control group. Mroczek-Sosnowska et al. (2017) evaluated nano-copper as an agent to minimize the problem of weak bones in broiler chickens, which is caused by a rapid body weight gain and imbalance between the increase in muscle mass and bone mass. Injection *in ovo* of copper nanoparticles at the beginning of embryogenesis resulted in a femoral bone, which was characterized by a higher weight and volume and by significantly greater resistance to fractures as compared to the control group. Additionally, *in ovo* application of copper nanoparticles at the stage of chicken embryo development poseses no threat of excessive copper accumulation in poultry organs like liver and spleen and muscles on the age at slaughter (Mroczek-Sosnowska et al. 2014).

Copper nanoparticles can also have a negative effect on poultry. Morsy et al. (2021) found that CuO nanoparticles supplemented to the diet of broiler chickens caused severe pathological alterations of muscle and different edible organs (muscle, heart, liver, spleen, and kidneys) which was correlated with increasing Cu dose from low − 5 mg/kg to high dose − 15 mg/kg body weight. DNA fragmentation was caused due to oxidative stress. No effect on growth performance and immune status was observed. Additionally, decreases in antibody titer of Avian Influenza and New Castle viruses was noted, which can lead to decreased disease resistance.

#### 4.3.2. Pigs

Gonzales-Eguia et al. (2009) confirmed beneficial effects of copper nanoparticles (50 mg/kg) on weaned piglets, which improved microelement bioavailability and reduced its fecal excretion, increased crude fat digestibility, and enhanced growth performance as compared to the control group supplemented with CuSO_4_. Additionally, nano-copper supplementation increased the level of serum IgG, γ-globulin, and total globulin protein, as well as superoxide dismutase activity.

#### 4.3.3. Rabbits

Copper nanoparticles supplemented to the diet of rabbits, improved feed conversion ratio, final body weight, performance index, increased activity of superoxide dismutase enzyme, enhanced intestinal microbiota (increase in the total bacterial count, whereas decrease in the population of ureolytic bacteria, *Escherichia coli* and *Clostridium* spp.) and increased hemoglobin content. Supplementation of CuNPs had no significant effect on Cu concentration in plasma and muscles, but the content of copper in liver and feces was significantly higher than in the control group (Refaie et al. 2015).

### 4.4. Gold (Au) nanoparticles

Gold nanoparticles are known to be used for various biomedical applications, such as biosensors, photothermal therapy, and drug delivery (Sembratowicz et al. 2016). There is little research into the use of gold nanoparticles in the veterinary medicine and livestock production, which is relatively innovative (Gangadoo et al. 2016; Hassanen et al. 2020). In the veterinary science, gold nanoparticles-based diagnostics is used for the enhanced detection of major pathogens and toxins in poultry and livestock (e.g., bacterial diseases like anthrax caused by *Bacillus anthracis*, brucellosis caused by *Brucella* spp., a viral disease – Newcastle disease, aflatoxicosis caused by aflatoxins produced by *Aspergillus* spp.) (Manhas et al. 2021).

The first experiments concern the use of gold nanoparticles in poultry nutrition. Sembratowicz et al. (2016) showed that gold nanoparticles supplied to drinking water of Ross 308 chickens at doses of 5, 10 and 15 mg/L were accumulated in the intestinal walls in a dose- and time-dependent manner. *In vitro* experiments also indicated that nano-gold present in the jejunum had a negative influence on the iron, calcium and potassium absorption. This may be the result of inhibition of the activity of transport proteins, blocking ion channels, or causing inflammation of the intestines leading to destruction of the intestinal villi. Hassanen et al. (2020) tested gold nanoparticles (5 and 15 mg/L of drinking water) on 90 one-day-old mixbred Cobb broiler chicken to evaluate their impact on growth performance, antioxidant levels and immune defense. Lower NPs dose had a positive effect on growth performance (average body weight and the feed conversion ratio) without any significant difference in immunological parameters, oxidative stress damage, pro-inflammatory cytokines, DNA fragmentation assay and histopathological organizations in some organs such as spleen, liver, bursa of fabricius, thymus, as compared with the control group.

### 4.5. Selenium (Se) nanoparticles

Selenium is known to play an important role in the antioxidant status of animals – it prevents oxidative stress (Sadeghian et al. 2012; Kojouri et al. 2020; Nabi et al. 2020; Han et al. 2021). Feed supplementation with selenium can improve not only the efficiency of the antioxidant system, but also can enhance disease resistance and nutritional quality of the livestock-based products (Amlan and Lalhriatpuii 2020; Shi et al. 2011a). Nano-selenium as a feed additive is mainly tested in poultry and ruminants. SeNPs administered to the poultry diet can enhance growth performance – feed conversion ratio, nutrient digestibility, growth rate, productivity, egg production, improve eggs and meat quality, increase microbial activity and gut microbial environment, and exert an antimicrobial activity (Nabi et al. 2020; Rana, 2021; Abdel-Moneim et al. 2022). In ruminants, nano-selenium can increase rumen fermentation and feed digestibility, enhance the body growth and weight gain, reduce oxidative stress and improve serum antioxidant enzymes (phospholipid hydroperoxide glutathione peroxidase, superoxide dismutase and catalase), improve the concentration of Se in serum, and maintain the animal’s reproductive physiology (Abdelnour et al. 2021). The role of selenium nanoparticles in animal feeding was described in detail by many researchers, for example Gangadoo et al. (2016), Amlan and Lalhriatpuii (2020), Nabi et al. (2020), Rana (2021), etc.

#### 4.5.1. Poultry

Selenium supplementation to poultry diet increases its antioxidative and immune properties (El-Deep et al. 2016; Gangadoo et al. 2020; Nabi et al. 2020). Gangadoo et al. (2018) demonstrated that selenium nanoparticles at a dose of 0.9 mg/kg of diet (among tested 0.3, 0.9 and 1.5 mg/kg) exhibited the best performance, improved gut health by increasing the population of beneficial bacteria, such as *Lactobacillus* and *Faecalibacterium prausnitzii* and short-chain fatty acids. El-Deep et al. (2016) showed that the addition of 0.3 mg of nano-selenium per kg of broiler chickens diet kept under high ambient temperature alleviated its negative effects such as decrease in body weight gain, feed intake, feed conversion ratio, weight of breast muscle and abdominal fat. Reduction in the content of malondialdehyde in liver and breast muscle was observed and increase in the Se and vitamin E content in breast muscle in the nano-selenium group. Selenium retention in liver and muscle was increased in a dose-dependent manner by dietary intake of nano-Se (0.3, 05, 1, and 2 mg/kg diet), but did not affect growth performance. However, meat quality, immune function, and oxidation resistance were improved by nano-Se levels ranging from 0.3 to 1 mg/kg diet (Cai et al. 2012).

The impact of *in ovo* injection of 5 or 10 ppb of SeNPs were explored on physiological responses, immunological status and growth performance of broiler chickens post hatching that were later supplemented or not with 10 ppb SeNPs/kg ration for 5 weeks (Ibrahim et al. 2020). The tested parameters increased for both examined routes of SeNPs delivery. The *in ovo* injection of SeNPs, along with their diet supplementation had improved body weight, body weight gain and feed conversion ratio. In addition, both delivery routes decreased serum triglycerides and malondialdehyde content, while high-density lipoprotein cholesterol and glutathione content were reduced, whereas glutathione reductase activity and immunoglobulin status were increased.

Dietary supplementation of growing quail with 0.2, 0.4, and 0.6 g/kg of diet with SeNPs increased body weight gain, and feed conversion ratio, while feed intake decreased compared to the control group with the highest effect recorded for 0.4 g/kg of diet. They significantly improved the plasma lipid profile, antioxidant enzymes activity, immunoglobulin G values, blood biochemistry, and bacterial environment of the intestine of quail (Alagawany et al. 2021).

Layer chickens group treated with nano-selenium (0.3 mg/kg – among tested doses 0.075, 0.15, 0.3 and 0.6 mg/kg of diet) increased relative weight gain and final body weight as compared to sodium selenite. Nano-Se treated groups had also higher content of Se in breast muscle and liver, increased activity of antioxidant enzymes – glutathione peroxidase, erythrocyte catalase and superoxide dismutase, increased serum biochemical (e.g., glucose, total protein, albumin, globulin) and hematological parameters (hemoglobin, Total Erythrocyte Count (TEC), Packed Cell Volume (PCV)) and finally increased cellular and humoral immunity (Mohapatra et al. 2014c). In addition to the ability of SeNPs to scavenge 2,2′-azino-bis(3-ethylbenzothiazoline-6-sulfonic acid) (ABTS) and 2,2-diphenyl-1-picrylhydrazyl (DPPH) radicals in a dose-dependent manner in a trial performed by Abdel-Moneim et al. (2022), they also possess considerable antimicrobial activity against Gram-positive, Gram-negative bacteria, *Candida* sp. and *Aspergillus* sp.

Feeding experiments on layer birds performed by Mohapatra et al. (2014b) showed that nano-selenium at a dose of 0.3 mg/kg of diet significantly increased Se content in breast muscle, liver, pancreas and feathers as compared to inorganic form – sodium selenite. Additionally, nano-selenium improved body weight, feed consumption ratio, antioxidant status (activity of glutathione peroxidase, erythrocyte catalase and superoxide dismutase), as well as cellular and humoral immunity. In comparison to sodium selenite, Meng et al. (2019) found that supplementing laying hens with nano-Se at 0.3 mg/kg of diet increased egg Se concentration and showed better retention in the body than the former. Qu et al. (2017) observed that SeNPs provided effective antioxidative protection against toxicity of laying hens with deoxynivalenol (DON). The supplementation of DON-contaminated basal diet (10 mg/kg DON) with 0.5 mg/kg SeNPs was able to improve glutathione peroxidase activity, total antioxidant status and egg production rate as well as decreased rate of soft-shelled or cracked eggs by increasing blood calcium level.

#### 4.5.2. Ruminants

Selenium nanoparticles can be also supplemented to the diet of sheep. Nano-selenium administered to the sheep diet, increased the resistance and defensive ability of the intrinsic immunity, as well as the neutrophil counts. Moreover, selenium nanoparticles showed better antioxidative effect than sodium selenite and were found to be less toxic and more bioactive than selenite (Sadeghian et al. 2012). In the work of Shi et al. (2011a), supplementation of nano-selenium to the basal diet of sheep improved feed utilization and rumen fermentation. The nutrient digestibility was higher for SeNPs supplemented at a dose of 0.3, 3 g/kg of diet than in the control group but lower for the dose of 6 g/kg of diet. With the increase of nano-selenium in the diet, ruminal volatile fatty acids concentration also increased. Nano-Se is also known to stimulate rumen microbial activity and enzyme activity. Supplementation of 4 g of nano-selenium (4 mg Se)/kg of diet in the work of Xun et al. (2012) decreased ruminal pH, ammonia N concentration and increased the total ruminal volatile fatty acids. Additionally, nano-selenium improved ruminal fermentation and increased feed conversion efficiency when compared to yeast–selenium.

Kojouri et al. (2020) tested nano-selenium in the diet of newborn lambs. SeNPs at a dose of 0.1 mg/kg of diet showed a significantly higher concentration of selenium in serum than for the control group, whereas the concentration of copper and zinc significantly decreased. SeNPs supplementation significantly improved the weight gain of lambs and increased the superoxide dismutase activity. The same effect was observed in the work of Yaghmaie et al. (2017) – nano-selenium (0.10 mg/kg of diet) reduced the oxidative stress (enhanced activity of blood glutathione peroxidase) and increased the lamb's weight gain. Selenium compound had no effects on Cu, Zn and Fe concentration in blood.

Han et al. (2021) showed that selenium nanoparticles supplemented to the diet of Holstein cows increased the concentration of Se in milk and activity of milk glutathione peroxidase when compared with sodium selenite, but there was no effect of selenium form on dry matter intake, as well as milk yield and composition.

#### 4.5.3. Rabbits

Sheiha et al. (2020) compared the effect of biologically and chemically synthesized selenium nanoparticles (25 and 50 mg/kg diets) on growth (daily body weight gain, and feed intake), carcass characteristics, oxidative (malondialdehyde, superoxide dismutase, glutathione, and catalase) and inflammatory parameters (interleukin 4, and interferon-gamma) of rabbits exposed to the thermal stress. Better results were observed for nano-silver produced by biological method. Additionally, these nanoparticles showed strong *in vitro* antibacterial activity against *Bacillus cereus*, *Listeria monocytogenes*, *Staphylococcus aureus*, *Escherichia coli*, *Salmonella typhimurium*, *Klebsiella pneumoniae* and *Pseudomonas aeruginosa*.

### 4.6. Calcium (Ca) nanoparticles

Calcium is a key element in the skeletal mineralization process (Matuszewski et al. 2020a). Reviews of Amlan and Lalhriatpuii (2020) and Matuszewski et al. (2020a) present examples on the effect of CaNPs on performance, immunity, and other biological traits in poultry. Since phosphorus is expensive among the mineral sources for poultry (broiler chickens), several studies proposed to replace its requirement as dicalcium phosphate by nano-form of calcium phosphate. Results from feeding experiments (body weight gain, and feed conversion ratio) showed that 50% dose of calcium phosphate nanoparticles can be used instead of the conventional 100% dose of dicalcium phosphate (Vijayakumar and Balakrishnan 2014; Samanta et al. 2019). Similarly, growth promoting effect was obtained by 1.75, 1.31, and 0.88% levels of calcium phosphate (Hassan et al. 2016), 0.12% levels of calcium phosphate-nanoparticles or supplementation of 2 to 10% of hydroxyapatite nanoparticles (Sohair et al. 2017) comparing to the control birds fed with conventional dicalcium phosphate (DCP). Nano-dicalcium phosphate supplemented to the diet of broilers, increased bone parameters such as tibia ash, Ca and P content when compared with conventional dicalcium phosphate (Mohamed et al. 2016).

Abo El-Maaty et al. (2021) showed that biosynthesized with marine seaweed – *Sargassum latifolium* extract calcium nanoparticles had a positive effect on egg and shell weight of laying hens, increased the level of serum Ca and P when compared with the control group. Addition of Ca carbonate nanoparticles to drinking water (1 g/L) of layers produced more eggshell strength and freshness indices (Wang et al. 2017). Neverthless, the egg production rate and blood Ca level were decreased by replacing 4.03% of Ca carbonate by 2.02%, 1.01%, 0.25% Ca carbonate nanoparticles that may be attributed to too much reduction in the Ca level in the layer hen's diet (Ganjigohari et al. 2018).

### 4.7. Chromium (Cr) nanoparticles

In broilers exposed to heat stress, the favourable effects of chromium (III) picolinate nanoparticles (NCrPic) applied at doses 500, 1000, and 1500 ppb were explored on growth performance, stress-related hormonal changes, serum levels of different immune biomarkers, and IFN-gene expression (Hamidi et al. 2022). Accordig to findings of Hamidi and his co-authors, lower body weight, daily weight gain, daily feed intake due to heat stress, and feed conversion ratio were all significantly improved by supplementation of NCrPic. NCrPic significantly lowered stress-elevated cortisol and immunoglobulin concentrations as well as upregulated the upregulation of IFN-expression. Similarly, in quail broilers, supplementation of nano-Cr at 800 µg/kg diet was effective in reducing the negative effects of physiological stress induced by dexamethazone. The performance and hematological parameters were not affected with nanoparticles-chromium, but they increased meat oxidative stability in stressed birds by reducing malondialdehyde concentration in the thigh muscle (Yarmohammadi et al. 2020). Neither the growth performance of birds nor the rate of egg production was affected by supplementation of chromium nanoparticles. However, the meat and egg quality, as well as tissue retention of different minerals was improved by their adminstration (Sirirat et al. 2013; Sathyabama and Jagadeeswaran 2016; Sathyabama et al. 2017). The conflicting results obtained from studies conducted on farm animals may be due to the use of different quantities and forms of Cr, as well as to differences in the means and time of use, age and species of the birds (Farag et al. 2017; Ognik et al. 2020).

### 4.8. Manganese (Mn) nanoparticles

Manganese in animal and poultry is responsible for proper bone formation and several biochemical processes (Matuszewski et al. 2020b). This micronutrient is required as a cofactor and activator of many enzymes, like galactosyltransferase, glutamine synthetase, agmatinase, arginase, pyruvate carboxylase, and superoxide dismutase to ensure appropriate growth and development (Avila et al. 2013). After nano-MnSO_4_ supplementation, tibia bone parameters such as tibia length, volume, breaking strength, diameter, and bone weight have been improved (Patra and Lalhriatpuii 2020). Jankowski et al. (2019) explored how different quantities of manganese (10, 50, and 100 mg/kg diet), either in the form of manganese oxide (MnO) or manganese nanoparticles (NPs-Mn_2_O_3_), affected growth performance, absorption, and accumulation of Mn, Zn, and Cu, antioxidant and immunological status in growing turkeys. The turkeys' development performance was not affected by reducing the amount of Mn in the feed from 100 to 50 or even 10 mg/kg, or by replacing MnO with NPs-Mn_2_O_3_. Interestingly, replacing MnO with NPs-Mn_2_O_3_ enhanced ileal Mn digestibility and reduced Cu buildup in the liver and breast muscle. Similar doses were used by Ognik et al. (2019) – they showed that regardless the dosage of Mn in turkey's diets, either the form of manganese – MnO or NPs-Mn_2_O_3_, Mn induced lipid oxidation reactions to the greatest extent. NPs-Mn_2_O_3_ reduced protein nitration more than MnO. They also concluded that the reduction of the Mn level, regardless of the form used, showed negative effect regarding the antioxidant activity that may be weakened and consequently induce oxidative processes in the cell. In broiler chickens, the use of NanoMn_2_O_3_ had no negative impact on development and growth, but reduced the excretion of Mn (Matuszewski et al. 2020b).

## 5. Concerns about the use of nanoparticles in animal nutrition

As shown in the present review, the supplementation of metal-containing nanoparticles in animal diets could be a promising option in the future. Encouraging results from recent studies on animals are the driving force for further investigations (Scott et al. 2018). But the potential hazard of a long-term application of nanoparticles as feed additives and their toxicological data are still unknown. Before nanoparticles can be recommended in animal nutrition, their toxicity and safety margins should be evaluated in animals tested for a long time (Amlan and Lalhriatpuii 2020). The small size of nanoparticles can affect their toxicity through easier cellular uptake, translocation in the animal’s body and retention (Gatoo et al. 2014; Exbrayat et al. 2015; Muralisankar et al. 2016; Conine and Frost 2017; Hill and Li 2017; Scott et al. 2018; Kumar and Bhattacharya 2019; Youssef et al. 2019; Gangadoo et al. 2020; Bidian et al. 2021). For example, *in vitro* findings showed that silver nanoparticles cause apoptosis and oxidative stress in cow granulosa cells, as well as alter the pattern of steroid hormone synthesis, as a result AgNPs may decrease the function and viability of ovarian cells (Tabandeh et al. 2022). Another main concern is the accumulation of these nanoparticles in the internal organs of treated animals (e.g., kidneys, liver), as well as in animal-derived products such as meat (Fondevila et al. 2009; Abdelsalam et al. 2019; Kumar and Bhattacharya 2019; Naz et al. 2021), which may be toxic for human beings (Exbrayat et al. 2015; Abdelsalam et al. 2019). Feeding animals with metallic/metal oxide nanoparticles requires understanding their mechanism of action to ensure safety for animals as well as further food quality assurance tests to confirm its safety for people (Hill and Li 2017; Hassanen et al. 2020; Awaad et al. 2021). A great amount of research is still required to support the safety of metal-containing nanoparticles application in animal nutrition, avoiding any harm to livestock, the environment, and human beings (Exbrayat et al. 2015; Scott et al. 2018).

## 6. Conclusions

In the present review it was shown that metal-containing nanoparticles, produced by physical, chemical, or biological methods can be used as beneficial feed additives. The supplementation of animal diet with elements such as copper, silver, zinc, gold, selenium, chromium, or calcium in nano-form has a positive effect on livestock and poultry performance, productivity, and health (especially antioxidant status and immunity). Nano-elements also proved to have antimicrobial activities against viral, bacterial (including drug resistant bacterial strains), parasitic, and fungal pathogens as well as effects on mycotoxins. They are proposed as an alternative to antibiotic growth promoters. Due to the inhibition of pathogens, nanoparticles are responsible for the intestinal health improvement. One of the main advantages of nanoparticles is their small size, thanks to which smaller doses can be administered in the animal's diet instead of bulk inorganic salts. At the same time, it is possible to prevent the excessive accumulation of these metals in waste, which results in the reduction of environmental damage. Although nanoparticles have many advantages there are also some obstacles in using them in animals. Small size of the nano-scale elements relates to their higher toxicity. Accumulation of metal-containing nanoparticles can induce cell death or can cause pathological changes and damage to the whole organism. Therefore, further very careful and precise toxicity studies are necessary before determining an appropriate, safe dose of dietary supplementation for livestock.
